# Supplementary material for: Individual retrotransposon integrants are differentially controlled by KZFP/KAP1-dependent histone methylation, DNA methylation and TET-mediated hydroxymethylation in naïve embryonic stem cells
Source: Epigenetics Chromatin. 2018 Feb 26;11:7. doi: 10.1186/s13072-018-0177-1 (PMC6389204; doi:10.1186/s13072-018-0177-1)
Supplement: Supplementary file 11 — Additional file 11. Pattern analysis. [file 13072_2018_177_MOESM11_ESM.zip › Patterns analysis/DataTables/extensions/Scroller/examples/index.html]

Scroller examples - Scroller examples


# Scroller example Scroller examples

Scroller is a virtual rendering plug-in for DataTables which allows large datasets to be drawn on
screen every quickly. What the virtual rendering means is that only the visible portion of the table
(and a bit to either side to make the scrolling smooth) is drawn, while the scrolling container gives
the visual impression that the whole table is visible. This is done by making use of the pagination
abilities of DataTables and moving the table around in the scrolling container DataTables adds to the
page. The scrolling container is forced to the height it would be for the full table display using an
extra element.

Scroller is initialised by simply including the letter `S` in the `domDT` for the table you want to have this
feature enabled on. Note that the `S` must come after the `t` parameter in
`domDT`.

Key features include:

- Speed! The aim of Scroller for DataTables is to make rendering large data sets fast
- Full compatibility with DataTables' deferred rendering for maximum speed
- Integration with state saving in DataTables (scrolling position is saved)
- Easy to use

### Examples

- Basic initialisation
- State saving
- Client-side data source (50,000 rows)
- Server-side processing (5,000,000
  rows)
- API

Please refer to the DataTables documentation for full
information about its API properties and methods.  
Additionally, there are a wide range of extras and
plug-ins which extend the capabilities of
DataTables.

DataTables designed and created by SpryMedia Ltd © 2007-2014  
DataTables is licensed under the MIT license.
